# Baltimore College of Dental Surgery—Spring Lectures

**Published:** 1842-12

**Authors:** 


***Baltimore College of Dental Surgery—Spring Lectures.***—In order to extend the usefulness of this Institution, by affording greater facilities to those who may wish to prepare themselves for the practice of Dental Surgery, the Faculty have determined to give a Spring Course of Lectures, and also to reduce the expenses of instruction.

The Spring course of 1843, will commence the second Monday in March next, and continue two months. The cost of tickets for this course, the matriculation included, will be sixty-five dollars. The price of tickets for the winter course has been reduced from thirty to twenty-five dollars each.

Candidates for graduation will be expected to attend one winter and one spring course, or two winter courses. One course of lectures however, in a respectable medical school is received as equivalent to one in the College of Dental Surgery. And, by a resolution of the Faculty, it has been determined that four years practice in dentistry shall be received as equivalent to a winter course in this institution.

By this new arrangement the expense of travelling, board and tuition will be greatly diminished.

Good board can be obtained here in private families at three dollars a week.

